# Development of the “National Asbestos Profile” to Eliminate Asbestos-Related Diseases in 195 Countries

**DOI:** 10.3390/ijerph18041804

**Published:** 2021-02-12

**Authors:** Diana Arachi, Sugio Furuya, Annette David, Alexander Mangwiro, Odgerel Chimed-Ochir, Kenneth Lee, Peter Tighe, Jukka Takala, Tim Driscoll, Ken Takahashi

**Affiliations:** 1 Asbestos Diseases Research Institute, Concord, NSW 2139, Australia; diana.arachi@adri.org.au; 2 Japan Occupational Safety and Health Research Center, Tokyo 204-0024, Japan; 2009aban@gmail.com; 3 Health Partners LLC, Tamuning 96913, Guam; amdavid@guam.net; 4 Secretariat of the Basel, Rotterdam and Stockholm Conventions, United Nations Environment Program, 1219 Geneva, Switzerland; alexander.mangwiro@un.org; 5 Department of Environmental Epidemiology, Institute of Industrial Ecological Sciences, University of Occupational and Environmental Health, Kitakyushu 807-8555, Japan; odgerel@med.uoeh-u.ac.jp; 6 Department of Anatomical Pathology, Concord Repatriation General Hospital, Concord, NSW 2139, Australia; Kenneth.lee@health.nsw.gov.au; 7 Asbestos Diseases Research Foundation, Concord, NSW 2139, Australia; peter.tighe08@gmail.com; 8 International Commission on Occupational Health, 20122 Milan, Italy; jstakala@gmail.com; 9 School of Public Health, The University of Sydney, Sydney, NSW 2006, Australia; tim.driscoll@sydney.edu.au; 10 University of Occupational and Environmental Health, Kitakyushu 807-8555, Japan

**Keywords:** asbestos, policy, World Health Organization, International Labor Organization, National Asbestos Profile, prevention, mesothelioma

## Abstract

Worldwide, 230,000+ people die annually from asbestos-related diseases (ARDs). The World Health Organization (WHO) recommends that countries develop a National Asbestos Profile (NAP) to eliminate ARDs. For 195 countries, we assessed the global status of NAPs (A: *bona fide* NAP, B: proxy NAP, C: relevant published information, D: no relevant information) by national income (HI: high, UMI: upper-middle, LMI: lower-middle, LI: low), asbestos bans (banned, no-ban) and public data availability. Fourteen (7% of 195) countries were category A (having a *bona fide* NAP), while 98, 51 and 32 countries were categories B, C and D, respectively. Of the 14 category-A countries, 8, 3 and 3 were LMI, UMI and HI, respectively. Development of a *bona fide* NAP showed no gradient by national income. The proportions of countries having a *bona fide* NAP were similar between asbestos-banned and no-ban countries. Public databases useful for developing NAPs contained data for most countries. Irrespective of the status of national income or asbestos ban, most countries have not developed a NAP despite having the potential. The global status of NAP is suboptimal. Country-level data on asbestos and ARDs in public databases can be better utilized to develop NAPs for globally eliminating ARDs.

## 1. Introduction

A recent Global Burden of Disease (GBD) study estimated that each year more than 230,000 people die from diseases caused by occupational exposure to asbestos [1]. In 2006, the World Health Organization (WHO) declared that the most efficient way to eliminate asbestos-related diseases (ARDs) is to stop using all types of asbestos [2]. The following year, the WHO and the International Labor Organization (ILO) jointly formulated the National Program for the Elimination of Asbestos-Related Diseases (NPEAD) [3,4] to assist countries in establishing their respective national programs.

The National Asbestos Profile (NAP) was annexed to the NPEAD as a template to support the development of country profiles consisting of 18 items related to legislation, asbestos use, ARD status and risk assessment. As such, the NAP is an internationally standardized instrument that is designed to define the baseline situation of a country and measure its progress towards eliminating ARDs. In 2014, the WHO reiterated the 2006 declaration and published the NAP for the second time. Of the 18 NAP items, four and six items are related to the status of asbestos use and ARDs, respectively. Information and data related to asbestos use and ARDs are thus essential for countries to develop a NAP.

Asbestos use is declining at the global level, but national situations range from “totally banned” to “mining and exporting raw asbestos,” or “manufacturing and/or using asbestos-containing products.” Almost 70 countries/territories have adopted asbestos bans to date [5], but this is skewed towards developed (i.e., *higher*-income) countries. Many developing (i.e., *lower*-income) countries have been slow to reduce, let alone ban, the use of asbestos [6]. When countries use asbestos, their country-level volume and rate of use correlate well with the subsequent disease burden and rates of ARD [7,8].

The national situations of ARDs also vary, with some countries having no data while others report data of irregular quality. Based on data reported by 83 member states of the WHO, the worldwide age-adjusted mortality rate of mesothelioma increased 5.4% annually from 1994 to 2008 [9]. An updated analysis continued to show a general increase of the age-adjusted mortality rate, based on data judged to be “reliable” from 59 member states [10]. A GBD study estimated an 82% increase in global mesothelioma deaths from 1990 to 2016 [11]. However, reliable data on mesothelioma are not available from developing countries that continue to use large amounts of asbestos [12].

The development of NAPs will not only enable countries to monitor progress towards ARD elimination but also encourage countries to learn from each other’s experiences and collectively promote the global elimination of ARDs. However, at present, there is no information on the global status of NAPs, let alone factors that encourage countries to develop (or inhibit countries from developing) their NAPs. Thus, the objective of this study was to assess the extent to which countries developed NAPs, or had the potential to do so, in relation to baseline factors such as national income status, asbestos bans and the availability of public data that can be used to develop a NAP.

## 2. Materials and Methods

We analyzed the status of information and data for 195 countries comprising 193 United Nations (UN) Member States [13] and two regional entities, Taiwan and Hong Kong. In our search of NAPs and related information, we explored the World Wide Web using English, German, French and Spanish. We also communicated with contacts of the coauthors, which included the current and former staff of international organizations, governments and non-government organizations. To determine the status of NAPs that corresponded to the countries, we applied the following criteria, which were ordinally categorized and mutually exclusive (Table 1):

When a document was identified as a *bona fide* NAP, a copy was obtained by downloading or requesting it from the concerned parties.

Two authors (DA, KT) rated the NAP statuses and thereby grouped the countries into four categories: A (country that has a *bona fide* NAP), B (country that does not have a *bona fide* NAP but has a proxy NAP), C (country that has neither a *bona fide* nor proxy NAP but has relevant published information) and D (country that has no relevant information). A disagreement between the two raters was reconciled by rechecking their ratings and, if the disagreement persisted, having a third author (SF) act as the tiebreaking rater to establish the final rating (there were three instances).

As basic characteristics, we grouped the analyzed countries as high income, upper-middle income, lower-middle income and low income based on the Income Classification of the World Bank [14]. Regions were based on the WHO region designation [15]. Regarding the status (banned or no-ban) and year of asbestos ban, we referred to the list of Current Asbestos Bans on the website of the International Ban Asbestos Secretariat [5].

To assess the availability of data that can be used to develop a NAP, we used the following: two sources of asbestos-related data, namely, (1) consumption of raw asbestos in the United States Geological Survey database (USGS) [16] and (2) import of asbestos-containing material, textiles and friction material in the United Nations International Trade Statistics Database (UN Comtrade) [17]; and four sources of disease-related data, namely, (1) reported mortality of mesothelioma or asbestosis in the WHO Mortality Database (MDB) [18], (2) estimated incidence of mesothelioma in the GBD studies [1], (3) estimated mortality of mesothelioma in the WHO Global Health Estimates database (GHE) [19] and (4) reported or estimated mortality of mesothelioma in the WHO Global Cancer Observatory (GCO) [20].

All data sources were publicly available. Microsoft Excel Version 16 (Microsoft Corporation, Washington DC, USA) was used to compile and analyze all data.

## 3. Results

Table 2 shows the basic characteristics of 195 countries grouped by NAP status. Of them, 14 (7%) had a *bona fide* NAP (category A), 98 (50%) did not have a *bona fide* NAP but had a proxy NAP (category B), 51 (26%) had neither a *bona fide* nor proxy NAP but had other relevant published information (category C), and 32 (16%) had no relevant published information (category D). Of the 14 countries that had a *bona fide* NAP (category A), most were LMI countries (*n* = 8), followed by UMI countries (*n* = 3) and HI countries (*n* = 3), as per the national income status. In terms of region, seven, four and three countries were in the Western Pacific, South East Asia and Europe, respectively. In terms of asbestos ban status, five countries had bans, while nine did not. The NAP categories with the highest proportion of countries by income status were B (78%), B (52%), C (33%) and D (41%) in the HI, UMI, LMI and LI categories, respectively. Of the 32 countries that had no relevant published information (category D), the greatest proportion corresponded to LI countries, followed sequentially by LMI, UMI and HI countries.

Of the 195 countries, 65 (33%) countries had asbestos bans and 130 (67%) countries did not. In the group of 65 asbestos-banned countries, 5 (8%) had a *bona fide* NAP, 51 (78%) had no *bona fide* NAP but had a proxy NAP, and 9 (14%) had neither a *bona fide* nor proxy NAP but had other relevant published information. No asbestos-banned country lacked relevant published information. In the group of 130 no-ban countries, 9 (7%) had a *bona fide* NAP, 47 (36%) had no *bona fide* NAP but had a proxy NAP, 42 (32%) had neither a *bona fide* nor proxy NAP but had other relevant published information, and 32 (25%) had no relevant published information.

**Figure 1:**
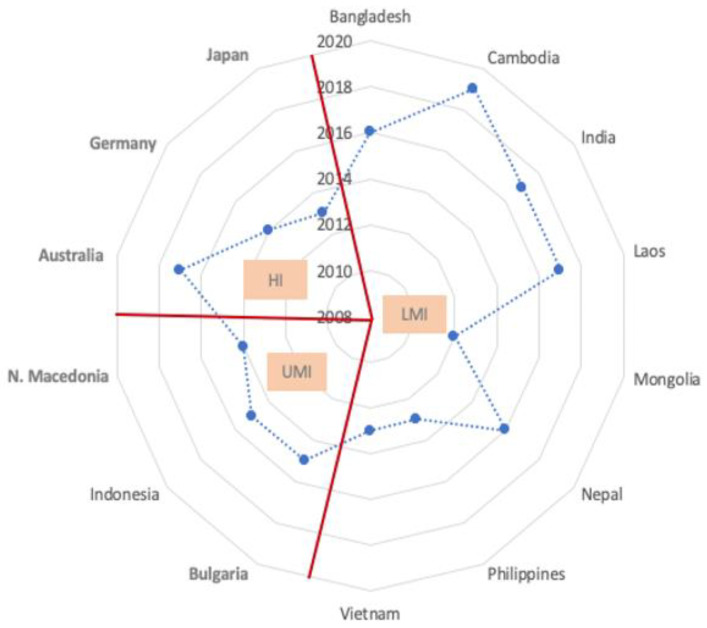
Countries that published a National Asbestos Profile, along with publication year and other characteristics.

Figure 1 shows 14 countries with a *bona fide* NAP by their year of NAP publication and national income category. The embedded table supplements information on the region, the status and year of asbestos ban, and the authoring group/organization. (Table 3) All NAPs were published in the 2010 decade. The five NAP-published countries that banned asbestos were Australia, Japan, Bulgaria, Germany and North Macedonia. Of them, North Macedonia published their NAP in the year of their asbestos ban; the other four countries published their NAPs 8–14 years after their ban. Multiple stakeholders (*n* = 9) were the most frequent authoring group, followed by government (*n* = 8) and non-government (*n* = 6) organizations (the total exceeds 14 due to some countries being counted in multiple categories). All NAPs were written in English or had an English version except for the NAP of North Macedonia, which did not have an English version.

Table 4 shows the relationship between the NAP category and the availability of data that can be used for a NAP. Asbestos data were available from two data sources: (1) the USGS database on raw asbestos consumption; and (2) the UN Comtrade data on asbestos-containing materials. The overall data availability was 85% (165/195) and 92% (179/195) of all countries, respectively. When stratified by NAP category, USGS data on raw asbestos consumption were available for 100% (14/14), 85% (83/98), 84% (43/51) and 78% (25/32) of category A, B, C and D countries, respectively. Similarly, UN Comtrade data on asbestos-containing materials were available for 100% (14/14), 93% (91/98), 90% (46/51) and 88% (28/32) of category A, B, C and D countries, respectively.

Disease data were available from four data sources: (1) the MDB data on reported mortality of mesothelioma or asbestosis; (2) the GBD data on the estimated incidence of mesothelioma; (3) the GHE data on estimated mortality of mesothelioma; and (4) the GCO data on reported or estimated mortality of mesothelioma. The overall data availability was 49% (96/195), 95% (186/195), 91% (178/195) and 69% (134/195) of all countries, respectively. When stratified by NAP category, data availability was generally better for categories A and B and worst for category D. For example, the MDB data on reported mortality of mesothelioma or asbestosis were available for 50% (7/14), 64% (63/98), 37% (19/51) and 22% (7/32) of countries in categories A, B, C and D, respectively.

Table A1 lists the 14 *bona fide* NAPs and their references. Table A2 summarizes the data availability for each country across all six databases. Table A3 outlines the original NAP according each item (I-1 to I-18) to public data sources that can be utilized. Data for legislation-related items (I-1, I-15, I-16) were generally not available from international sources and thus needed to be sought from national sources. Data for asbestos-related items (I-2 to I-5) were generally available from the international databases mentioned above. Data for disease-related items (I-9 to I-12) were available from the international databases mentioned above. Although data for I-17 were generally not available from any source for most countries, data for I-18 were available in PubMed. Data for risk assessment (I-6 to I-8, I-13, I-14) were sometimes available from national sources.

## 4. Discussion

A total of 14 (7% of 195) countries developed *bona fide* NAPs (category A). The development of a *bona fide* NAP showed no gradient by national income: LMI countries comprised the highest proportion (16%) of countries that published a *bona fide* NAP, followed by UMI (6%) and HI (5%) countries, with no *bona fide* NAP developed by an LI country to date. At the opposite extreme, 32 (16% of 195) countries had no relevant published information (category D), and this showed a gradient with the national income category: LI countries comprised the highest proportion with no relevant published information, followed sequentially by LMI, UMI and HI countries. Furthermore, a comparatively poorer status of NAPs (i.e., categories C and D combined) correlated with lower national income. Therefore, our study demonstrated that although the NAP status was generally related to the national income status, the development of a *bona fide* NAP was unrelated to the national income status in all but LI countries.

Ninety-eight (50% of 195) countries did not have a *bona fide* NAP but did have a proxy NAP (category B). As a proxy NAP was defined as being compatible in content with a *bona fide* NAP, they should be similar in their resources and information. It is thus reasonable to assume that the 98 countries (in category B) had the full potential (i.e., resources and information) to develop a *bona fide* NAP. A further 51 (26% of 195) countries had neither a *bona fide* nor proxy NAP but had other relevant published information (category C) and thus could have had *some* potential to develop a *bona fide* NAP. In effect, a combined 149 (76% of 195) countries had some or full potential to develop a NAP.

Two sources of data for asbestos and four sources of data for ARDs were available to develop a NAP. Importantly, these sources contained data for most of the countries, and there was a minimal gradient of data availability across the NAP categories (Table 4). A notable exception was the WHO MDB; this database compiles data *reported* by countries, and fewer than 50% of the countries were covered for mesothelioma mortality. However, estimated data can compensate for the lack of reported data, provided that a country indicates the nature of data that are incorporated in the NAP. The low number (*n* = 14) and proportion (7%) of all countries that had developed a *bona fide* NAP should thus be viewed in consideration of the wide availability of country-level data on asbestos and ARDs.

Mesothelioma is widely accepted as an indicator disease caused by asbestos exposure, with at least 80% specificity [21]; it is thus a key item for a NAP. Although more than 50% of the countries did not report mesothelioma deaths to the WHO, estimates are currently available for more than 90% of the countries in the two data sources (Table 4). Although many *lower*-income countries started to consume asbestos recently, some of them may not have reached the generally accepted latency period of 30–40 years for mesothelioma [21]. Moreover, many *lower*-income countries have not yet acquired the technology/infrastructure to diagnose and report mesothelioma and thus may be “missing” the disease burden. It is important for countries lacking mesothelioma data to utilize these estimates; that said, it is also important that they understand the method of imputation to derive the estimates (e.g., asbestos use is commonly imputed) as well as their limitations [10].

The regional distribution of the 14 NAP-published countries was skewed, with the majority situated in Asia (seven in the Western Pacific and four in South-East Asia), three in Europe and none in the Americas, Africa or Eastern Mediterranean (Table 3). The regional preponderance may have been caused by a combination of “pull” and “push” factors. Possible pull factors are that Europe is the known current center of the ARD burden [22], and Asia has been implicated as the future “center” [23] of this burden due to its current heavy use of asbestos. Possible push factors include the WHO/ILO partnerships (e.g., the 2010 Parma Declaration on Environment and Health specified establishment of NPEAD for the member states of WHO-Europe [24]) and grass-roots initiatives on advocacy and technology transfer (e.g., the Asian Asbestos Initiative) [25]. On the other hand, pro-asbestos lobbies influence asbestos use in industrializing countries [6] and may present “opposing” factors. All these factors will impact the development (or lack thereof) of a NAP.

In terms of the relationship between the NAP category and asbestos-ban status, the proportion of countries having a bona fide NAP was similarly low in asbestos-banned (8% or 5/65) and no-ban (7% or 9/130) countries. The lack of association between the status of NAP and asbestos-ban is a positive finding because the acceptance of a NAP should not be limited to either asbestos-banned or no-ban countries. The NAP is an effective tool to outline the national situation on asbestos and ARDs. The development of a NAP benefits no-ban countries by informing the progress towards the adoption of an asbestos ban and benefits asbestos-banned countries by informing the progress in reducing exposure to in situ asbestos and transitioning to an asbestos-free society.

Most (56 [86%] of 65) of the asbestos-banned countries had *either* a *bona fide* NAP or a proxy NAP (i.e., categories A and B combined), while more than half (74 [57%] of 130) of no-ban countries had *neither* of the two (i.e., categories C and D combined). Asbestos-banned countries may build a “knowledge base” of experience, information and data, which accumulate over the various phases of asbestos use, ban and post-ban. This knowledge base is likely to be documented in various forms, including laws, regulations, advisories, status reports and official statistics. These countries can thus capitalize on the abundant experience and resources to develop their NAPs. In contrast, no-ban countries may have a less extensive “knowledge base”, fewer resources and less experience.

For the 14 existing NAPs, multiple stakeholder authorship was common, and government representatives were often involved, with others or on their own. This finding corroborates the importance of employing multidisciplinary expertise with government representation in developing a NAP. Governments routinely collect information on industry and the labor force and collect (albeit to a lesser extent) surveillance data on asbestos and ARDs. General information on industry and the labor force constitutes baseline information and may be documented in the NAP to provide a national context. However, the highest priority should be given to incorporating national surveillance data on asbestos and ARDs. It is also important to observe that an equal disease incidence in men and women, rather than higher incidence in men due to occupational exposure to asbestos, could also alert countries to potential environmental exposure. Future studies are needed to review the use of ARD database information from this perspective.

The major limitations of this study are as follows: (1) We assessed the global status of the development of NAPs, not their utilization. For example, the NAP can be used to further develop a national action plan. Such a theme, however, is fundamentally different and warrants a separate study. (2) We cannot rule out the possibility that we missed identifying an existing *bona fide* NAP. (3) Our authors were involved in developing several NAPs (SF for the NAP of Japan; PT for the NAP of Australia; KT for the NAPs of Japan, Vietnam and Australia); although this experience may have added perspective and insight to the present work, we may not have been able to eliminate bias in judging a NAP as *bona fide* or not. The scope of this study is limited to mesothelioma and asbestosis and databases that use reported and/or estimated mortality. We highlight usable data sources from credible organizations that can be used to help and inform future NAPs. Despite being useful as an indicator of the asbestos burden, any database that uses estimates or country-level proxy data as a method has limitations. A strength of this study is that we were able to analyze the status of NAP development for most countries of the world and offer a framework for more countries to develop a NAP.

## 5. Conclusions

In conclusion, the global status of NAPs is suboptimal. Irrespective of the status of national income or asbestos ban, most countries of the world have not developed a NAP despite having the potential (i.e., resources and information) to do so. Among the few countries that have developed a *bona fide* NAP, LMI and UMI countries outnumber HI countries. Country-level data on asbestos and ARDs in public databases can be utilized to develop a NAP. All countries should develop their NAP and use it to monitor progress towards eliminating ARDs, learn from the experience of other countries and contribute to promoting the global elimination of ARDs.

## Figures and Tables

**Table 1:** Country and Document Categories.

Country Category	Document Category	Document Description
A	“*bona fide* NAP”	a single document that describes the national situation of asbestos and asbestos-related diseases (ARDs) in adherence to the NAP format published by the WHO/ILO^3^
B	“proxy NAP”	a single document or multiple documents that describe the national situation of asbestos and ARDs but does (do) not satisfy the criterion for a *bona fide* NAP; * includes government statements and/or decrees, scientific articles and third-party organization reports
C	“relevant published information”	information that does not satisfy the criteria for a *bona fide* or proxy NAP but refers to asbestos and/or ARDs; includes online information on asbestos as part of wider occupational health and safety policies, toxic chemical waste management policies, ARD case studies and media releases on asbestos and/or ARDs
D	“no relevant information”	status that lacked any of the above

* A proxy NAP was defined to be compatible in content with a *bona fide* NAP without satisfying the criterion of adhering to the NAP format published by the WHO/ILO.

**Table 2:** Basic characteristics of analyzed countries by the status of their National Asbestos Profile (NAP).

Country Category	Number of Countries	Income Category ^1^			Region							Status of Asbestos Ban	
		HI	UMI	LMI	LI	Western Pacific	South East Asia	Europe	Americas	Africa	East Mediterranean	Banned	No-Ban
A: Countries that have bona fide NAP ^2^	14	3	3	8	0	7	4	3	0	0	0	5	9
	(7%)	(5%)	(6%)	(16%)	(0%)	(28%)	(36%)	(6%)	(0%)	(0%)	(0%)	(8%)	(7%)
B: Countries that do not have bona fide NAP but have proxy NAP ^3^	98	49	28	15	6	17	4	39	14	12	12	51	47
	(50%)	(78%)	(52%)	(31%)	(21%)	(68%)	(36%)	(72%)	(40%)	(26%)	(57%)	(78%)	(36%)
C: Countries that have neither bona fide or proxy NAP but have other relevant published information	51	10	14	16	11	2	2	8	13	19	7	9	42
	(26%)	(16%)	(26%)	(33%)	(38%)	(8%)	(18%)	(15%)	(37%)	(40%)	(33%)	(14%)	(32%)
D: Countries with no relevant published information	32	1	9	10	12	1	1	4	8	16	2	0	32
	(16%)	(2%)	(17%)	(20%)	(41%)	(4%)	(9%)	(7%)	(23%)	(34%)	(10%)	(0%)	(25%)
All countries	195	63	54	49	29	27	11	54	35	47	21	65	130
	(100%)	(100%)	(100%)	(100%)	(100%)	(100%)	(100%)	(100%)	(100%)	(100%)	(100%)	(100%)	(100%)

^1^ HI: high income; UMI: upper-middle income; LMI: lower-middle income; LI: low-income based on the World Bank Income Classification. ^2^ See text for exact definition of bona fide NAP. ^3^ See text for exact definition of proxy NAP.

**Table 3:** Countries that published a National Asbestos Profile, Along With Publication Year and Other Characteristics.

Country	NAP Publication Year	National Income Category ^1^	Region ^2^	Asbestos Ban	Authors ^3^
Australia	2017	HI	WP	2003	G
Bangladesh	2016	LMI	SEA	No-ban	NG
Bulgaria	2015	UMI	EUR	2005	G
Cambodia	2019	LMI	WP	No-ban	G, MS
Germany	2014	HI	EUR	2005	G
India	2017	LMI	SEA	No-ban	NG
Indonesia	2015	UMI	SEA	No-ban	NG, MS
Japan	2013	HI	WP	2007	NG, MS
Laos	2017	LMI	WP	No-ban	G, MS
North Macedonia	2014	UMI	EUR	2014	NG, MS
Mongolia	2012	LMI	WP	No-ban	G, MS
Nepal	2016	LMI	SEA	No-ban	NG, MS
Philippines	2013	LMI	WP	No-ban	G, MS
Vietnam	2013	LMI	WP	No-ban	G, MS

^1^ HI: High Income; UMI: Upper-Middle Income; LMI: Lower-Middle Income; ^2^ WPR: Western Pacific; SEAR: South-East Asia; EUR: Europe; ^3^ G: Government; NG: Non-Government; MS: Multiple Stakeholders.

**Table 4:** Availability of data that can be used for NAPs in public databases.

Country Category	Data Availability					
	*Asbestos Data*		*Disease Data*			
	Consumption of Raw Asbestos < USGS ^1^ > (Row %)	Asbestos-Containing Material, Cement, Textiles or Friction Material < UN Comtrade ^2^ > (row %)	Reported Mortality of Mesothelioma or Asbestosis ^3^ < WHO MDB ^3^ > (Row%)	Estimated Incidence of Mesothelioma < GBD ^4^ > (Row %)	Estimated Mortality of Mesothelioma < WHO GHE ^5^ > (Row %)	Reported or Estimated Mortality of Mesothelioma < WHO GCO ^6^ > (Row%)
A (*n* = 14)	14 (100%)	14 (100%)	7 (50%)	14 (100%)	14 (100%)	13 (93%)
B (*n* = 98)	83 (85%)	91 (93%)	63 (64%)	91 (93%)	87 (89%)	77 (79%)
C (*n* = 51)	43 (84%)	46 (90%)	19 (37%)	49 (96%)	47 (92%)	27 (53%)
D (*n* = 32)	25 (78%)	28 (88%)	7 (22%)	32 (100%)	30 (94%)	17 (53%)
All countries (*n* = 195)	165 (85%)	179 (92%)	96 (49%)	186 (95%)	178 (91%)	134 (69%)

^1^ From the United States Geological Survey, Asbestos Statistics and Information: available data for 1920–2017 used. ^2^ The UN International Trade Statistics Database was tabulated for available data for asbestos-containing materials, Comtrade code 681,140 (asbestos cement; articles thereof; years available 2017–2019) or Comtrade code 6812 (fabricated asbestos fibers; mixtures with a basis of asbestos or of asbestos and magnesium carbonate; articles of such mixtures or of asbestos; years available 1996–2018) or Comtrade code 681,320 (friction material and articles thereof not mounted; for brakes, clutches or the like, with a basis of asbestos; years available 2007–2018): available data for timeframe 1996–2019 used. ^3^ From the WHO Mortality Database: available data for ICD-10 Code C45 Mesothelioma or ICD-10 Code J61 Pneumoconiosis due to asbestos and other mineral fibers (asbestosis) and available data for timeframe 1994–2017 used. ^4^ From the Global Burden of Disease studies: available data for 2017 used. ^5^ From the WHO Global Health Estimates: available data for 2016 used. Countries with poor quality data were counted as countries with available data. See GHE website for a description of poor-quality data. ^6^ From the WHO Global Cancer Observatory: available data for 2018 used. Note that historical data not available due to differences in estimation methods.

## Data Availability

Data is available on request.

## Appendix A

**Table A1:** List of *bona fide* National Asbestos Profiles and their References.

Country	Reference
Australia	Asbestos Safety and Eradiation Agency. 2017. *National Asbestos Profile for Australia*. Available online: https://www.asbestossafety.gov.au/sites/default/files/documents/2017-12/ASEA_National_Asbestos_Profile_interactive_Nov17.pdf (accessed on 11 January 2021). [26]
Bangladesh	Bangladesh Occupational Safety, Health and Environment Foundation. 2016. *National Asbestos Profile of Bangladesh*. Obtained via personal communication: 20 April 2020. [27]
Bulgaria	Vangelova, K.; Dimitrova, S.; Dimitrova, I. 2015. National Asbestos Profile of Bulgaria. Available online: https://ncpha.government.bg/files/National%20Asbestos%20Profile_Bulgaria_2015-en.pdf (accessed on 11 January 2021). [28]
Cambodia	Ministry of Labor and Vocational Training. 2019. *Cambodia National Asbestos Profile*. Personal Communication, 2020. [29]
Germany	Federal Institute for Occupational Health and Safety. 2014. *National Asbestos Profile for Germany*. Available online: https://www.baua.de/EN/Service/Publications/Report/Gd80.pdf?__blob=publicationFile&v=8 (accessed on 11 January 2021). [30]
India	People’s Training & Research and Centre. 2017. India: National Asbestos Profile. Available online: https://amrc.org.hk/sites/default/files/NAP%20India.pdf (accessed on 11 January 2021). [31]
Indonesia	Indonesia Ban Asbestos Network. 2017. *National Asbestos Profile Indonesia*. Obtained via personal communication: 28 April 2020. [32]
Japan	Furuya, S.; Takahashi, K.; Mohaved, M.; Jiang, Y. 2013. *National Asbestos Profile of Japan*. Available online: https://www.researchgate.net/publication/237839114_National_Asbestos_Profile_of_Japan_-_Based_on_the_National_Asbestos_Profile_by_the_ILO_and_the_WHO (accessed on 11 January 2021) [33]
Laos	Laos Ministry of Industry and Commerce. 2017. *National Asbestos Profile of Laos*. Personal Communication, 2020. [34]
North Macedonia	Institute of Occupational Health of the Republic of Macedonia. 2014. *National Asbestos Profile for the Republic of Macedonia*. Personal Communication, 2020. [35]
Mongolia	Health Sciences University of Mongolia. 2012. *National Asbestos Profile of Mongolia*. Personal Communication, 2020. [36]
Nepal	Sah, R.C. 2016. National Asbestos Profile of Nepal. Available online: http://anroev.org/aban/wp-content/uploads/2016/10/National-Abestos-Profile-of-Nepal.pdf (accessed on 11 January 2021). [37]
Philippines	Republic of Philippines, Environmental and Occupational Health Office. 2013. National Asbestos Profile Philippines. Available online: https://www.informea.org/en/national-asbestos-profile-nap-philippines (accessed on 11 January 2021). [38]
Vietnam	Pham, V.H.; Tran, T.N.L.; Le, G.V.; Movahed, M.; Jiang, Y.; Pham, N.H.; Ogawa, H; Takahashi, K. Asbestos and asbestos-related diseases in Vietnam: In reference to the International Labor Organization/World Health Organization National Asbestos Profile. *Saf. Health Work.* **2013**, *4,* 117–121. doi:10.1016/j.shaw.2013.04.002. [39]

**Table A2:** Availability of Asbestos and Asbestos-Related-Diseases Data by Country and NAP Country Category.

					Asbestos Data		Disease Data			
	Countries ^1^	World Bank Income Group ^2^	Asbestos Ban Status ^3^	NAP Country Category ^4^	Consumption of Raw Asbestos (USGS) ^5^	Asbestos-Containing Material (UN Comtrade) ^6^	Reported Mortality of Mesothelioma or Asbestosis (WHO MDB) ^7^	Estimated Incidence of Mesothelioma (GBD) ^8^	Estimated Mortality of Mesothelioma (WHO GHE) ^9^	Reported or estimated Mortality of Mesothelioma (WHO GCO) ^10^
1	Afghanistan	LI	No-Ban	C	Yes	No	No	Yes	Yes	Yes
2	Albania	UMI	No-Ban	C	Yes	Yes	No	Yes	Yes	Yes
3	Algeria	LMI	Ban	C	Yes	Yes	No	Yes	Yes	Yes
4	Andorra	HI	No-Ban	C	No	Yes	No	Yes	No	No
5	Angola	LMI	No-Ban	D	Yes	Yes	No	Yes	Yes	Yes
6	Antigua and Barbuda	HI	No-Ban	D	Yes	Yes	No	Yes	No	No
7	Argentina	UMI	Ban	B	Yes	Yes	Yes	Yes	Yes	Yes
8	Armenia	UMI	No-Ban	D	Yes	Yes	Yes	Yes	Yes	Yes
9	Australia	HI	Ban	A	Yes	Yes	Yes	Yes	Yes	Yes
10	Austria	HI	Ban	B	Yes	Yes	Yes	Yes	Yes	Yes
11	Azerbaijan	UMI	No-Ban	C	Yes	Yes	No	Yes	Yes	Yes
12	Bahamas	HI	No-Ban	C	Yes	Yes	Yes	No	Yes	No
13	Bahrain	HI	Ban	B	No	Yes	Yes	Yes	Yes	Yes
14	Bangladesh	LMI	No-Ban	A	Yes	Yes	No	Yes	Yes	Yes
15	Barbados	HI	No-Ban	B	Yes	Yes	Yes	Yes	Yes	No
16	Belarus	UMI	No-Ban	D	Yes	Yes	No	Yes	Yes	Yes
17	Belgium	HI	Ban	B	Yes	Yes	Yes	Yes	Yes	Yes
18	Belize	UMI	No-Ban	D	Yes	Yes	Yes	Yes	Yes	No
19	Benin	LMI	No-Ban	D	Yes	Yes	No	Yes	Yes	Yes
20	Bhutan	LMI	No-Ban	C	Yes	Yes	No	Yes	Yes	No
21	Bolivia	LMI	No-Ban	B	Yes	Yes	Yes	Yes	Yes	Yes
22	Bosnia and Herzegovina	UMI	No-Ban	B	Yes	Yes	Yes	Yes	Yes	Yes
23	Botswana	UMI	No-Ban	C	Yes	Yes	No	Yes	Yes	No
24	Brazil	UMI	Ban	B	Yes	Yes	Yes	Yes	Yes	Yes
25	Brunei	HI	Ban	C	Yes	Yes	No	Yes	Yes	No
26	Bulgaria	UMI	Ban	A	Yes	Yes	Yes	Yes	Yes	Yes
27	Burkina Faso	LI	No-Ban	D	No	Yes	No	Yes	Yes	Yes
28	Burundi	LI	No-Ban	D	Yes	Yes	No	Yes	Yes	Yes
29	Cambodia	LMI	No-Ban	A	Yes	Yes	No	Yes	Yes	Yes
30	Cameroon	LMI	No-Ban	D	Yes	Yes	No	Yes	Yes	No
31	Canada	HI	Ban	B	Yes	Yes	Yes	Yes	Yes	Yes
32	Cape Verde	LMI	No-Ban	C	No	Yes	No	Yes	Yes	No
33	Central African Republic	LI	No-Ban	D	No	Yes	No	Yes	Yes	No
34	Chad	LI	No-Ban	C	Yes	No	No	Yes	Yes	No
35	Chile	HI	Ban	B	Yes	Yes	Yes	Yes	Yes	Yes
36	China	UMI	No-Ban	B	Yes	Yes	No	Yes	Yes	Yes
37	Colombia	UMI	Ban	B	Yes	Yes	Yes	Yes	Yes	Yes
38	Comoros	LMI	No-Ban	D	No	Yes	No	Yes	Yes	No
39	Congo–Brazzaville	LMI	No-Ban	D	Yes	Yes	No	Yes	Yes	Yes
40	Congo–Kinshasa (DR Congo)	LI	No-Ban	C	Yes	No	No	Yes	No	Yes
41	Costa Rica	UMI	No-Ban	B	Yes	Yes	Yes	Yes	Yes	No
42	Côte d’Ivoire	LMI	No-Ban	C	Yes	Yes	No	Yes	Yes	Yes
43	Croatia	HI	Ban	B	Yes	Yes	Yes	Yes	Yes	Yes
44	Cuba	UMI	No-Ban	C	Yes	Yes	Yes	Yes	Yes	Yes
45	Cyprus	HI	Ban	B	Yes	Yes	Yes	Yes	Yes	Yes
46	Czech Republic	HI	Ban	B	Yes	Yes	Yes	Yes	Yes	Yes
47	Denmark	HI	Ban	B	Yes	Yes	Yes	Yes	Yes	Yes
48	Djibouti	LMI	Ban	C	Yes	No	No	Yes	Yes	No
49	Dominica	UMI	No-Ban	D	Yes	Yes	No	Yes	Yes	No
50	Dominican Republic	UMI	No-Ban	D	Yes	Yes	Yes	Yes	Yes	Yes
51	Ecuador	UMI	No-Ban	B	Yes	Yes	Yes	Yes	Yes	Yes
52	Egypt	LMI	Ban	B	Yes	Yes	Yes	Yes	Yes	Yes
53	El Salvador	LMI	No-Ban	D	Yes	Yes	Yes	Yes	Yes	No
54	Equatorial Guinea	UMI	No-Ban	D	No	No	No	Yes	Yes	No
55	Eritrea	LI	No-Ban	D	Yes	No	No	Yes	Yes	Yes
56	Estonia	HI	Ban	B	Yes	Yes	Yes	Yes	Yes	Yes
57	Eswatini Swaziland	LMI	No-Ban	C	Yes	Yes	No	Yes	Yes	No
58	Ethiopia	LI	No-Ban	C	Yes	Yes	No	Yes	Yes	Yes
59	Fiji	UMI	No-Ban	B	Yes	Yes	Yes	Yes	No	No
60	Finland	HI	Ban	B	Yes	Yes	Yes	Yes	Yes	Yes
61	France	HI	Ban	B	Yes	Yes	Yes	Yes	Yes	Yes
62	Gabon	UMI	Ban	C	Yes	Yes	No	Yes	Yes	No
63	Gambia	LI	No-Ban	C	No	Yes	No	Yes	Yes	No
64	Georgia	UMI	No-Ban	C	Yes	Yes	Yes	Yes	Yes	Yes
65	Germany	HI	Ban	A	Yes	Yes	Yes	Yes	Yes	Yes
66	Ghana	LMI	No-Ban	B	Yes	Yes	No	Yes	Yes	Yes
67	Greece	HI	Ban	B	Yes	Yes	Yes	Yes	Yes	Yes
68	Grenada	UMI	No-Ban	C	No	Yes	Yes	Yes	No	No
69	Guatemala	UMI	No-Ban	C	Yes	Yes	Yes	Yes	Yes	No
70	Guinea	LI	No-Ban	D	Yes	Yes	No	Yes	Yes	No
71	Guinea-Bissau	LI	No-Ban	D	No	Yes	No	Yes	Yes	No
72	Guyana	UMI	No-Ban	C	Yes	Yes	Yes	Yes	Yes	No
73	Haiti	LI	No-Ban	C	Yes	No	No	Yes	Yes	Yes
74	Honduras	LMI	Ban	C	Yes	Yes	No	Yes	Yes	Yes
75	Hong Kong ^1^	HI	No-Ban	B	Yes	Yes	Yes	No	No	No
76	Hungary	HI	Ban	B	Yes	Yes	Yes	Yes	Yes	Yes
77	Iceland	HI	Ban	B	Yes	Yes	Yes	Yes	Yes	Yes
78	India	LMI	No-Ban	A	Yes	Yes	No	Yes	Yes	Yes
79	Indonesia	UMI	No-Ban	A	Yes	Yes	No	Yes	Yes	Yes
80	Iran	UMI	No-Ban	B	Yes	Yes	Yes	Yes	Yes	Yes
81	Iraq	UMI	Ban	B	Yes	No	Yes	Yes	Yes	Yes
82	Ireland	HI	Ban	B	Yes	Yes	Yes	Yes	Yes	Yes
83	Israel	HI	Ban	B	Yes	Yes	Yes	Yes	Yes	Yes
84	Italy	HI	Ban	B	Yes	Yes	Yes	Yes	Yes	Yes
85	Jamaica	UMI	No-Ban	B	Yes	Yes	Yes	Yes	Yes	Yes
86	Japan	HI	Ban	A	Yes	Yes	Yes	Yes	Yes	Yes
87	Jordan	UMI	Ban	B	Yes	Yes	Yes	Yes	Yes	Yes
88	Kazakhstan	UMI	No-Ban	B	Yes	Yes	Yes	Yes	Yes	Yes
89	Kenya	LMI	No-Ban	B	Yes	Yes	No	Yes	Yes	Yes
90	Kiribati	LMI	No-Ban	B	No	Yes	No	Yes	Yes	No
91	Kuwait	HI	Ban	C	Yes	Yes	Yes	Yes	Yes	Yes
92	Kyrgyzstan	LMI	No-Ban	D	Yes	Yes	Yes	Yes	Yes	Yes
93	Laos	LMI	No-Ban	A	Yes	Yes	No	Yes	Yes	Yes
94	Latvia	HI	Ban	B	Yes	Yes	Yes	Yes	Yes	Yes
95	Lebanon	UMI	No-Ban	B	Yes	Yes	No	Yes	Yes	Yes
96	Lesotho	LMI	No-Ban	B	No	Yes	No	Yes	Yes	Yes
97	Liberia	LI	No-Ban	B	Yes	No	No	Yes	Yes	Yes
98	Libya	UMI	No-Ban	B	Yes	Yes	No	Yes	Yes	Yes
99	Liechtenstein	HI	Ban	B	No	No	No	No	No	No
100	Lithuania	HI	Ban	B	Yes	Yes	Yes	Yes	Yes	Yes
101	Luxembourg	HI	Ban	C	Yes	Yes	Yes	Yes	Yes	Yes
102	Madagascar	LI	No-Ban	C	Yes	Yes	No	Yes	Yes	Yes
103	Malawi	LI	No-Ban	C	Yes	Yes	No	Yes	Yes	No
104	Malaysia	UMI	No-Ban	B	Yes	Yes	No	Yes	Yes	Yes
105	Maldives	UMI	No-Ban	C	Yes	Yes	Yes	Yes	Yes	No
106	Mali	LI	No-Ban	D	Yes	Yes	No	Yes	Yes	Yes
107	Malta	HI	Ban	B	Yes	Yes	Yes	Yes	Yes	Yes
108	Marshall Islands	UMI	No-Ban	B	No	No	No	Yes	No	No
109	Mauritania	LMI	No-Ban	D	No	Yes	No	Yes	Yes	No
110	Mauritius	HI	Ban	B	Yes	Yes	Yes	Yes	No	No
111	Mexico	UMI	No-Ban	B	Yes	Yes	Yes	Yes	Yes	Yes
112	Micronesia	LMI	No-Ban	B	No	Yes	No	Yes	Yes	No
113	Moldova	LMI	No-Ban	C	Yes	Yes	Yes	Yes	Yes	Yes
114	Monaco	HI	Ban	B	No	No	No	No	No	No
115	Mongolia	LMI	No-Ban	A	Yes	Yes	Yes	Yes	Yes	Yes
116	Montenegro	UMI	No-Ban	C	Yes	Yes	Yes	Yes	Yes	No
117	Morocco	LMI	No-Ban	C	Yes	Yes	Yes	Yes	Yes	Yes
118	Mozambique	LI	Ban	B	Yes	Yes	No	Yes	Yes	Yes
119	Myanmar	LMI	No-Ban	D	Yes	Yes	No	Yes	Yes	Yes
120	Namibia	UMI	No-Ban	C	Yes	Yes	No	Yes	Yes	Yes
121	Nauru	HI	No-Ban	B	No	No	No	No	No	No
122	Nepal	LMI	No-Ban	A	Yes	Yes	No	Yes	Yes	Yes
123	Netherlands	HI	Ban	B	Yes	Yes	Yes	Yes	Yes	Yes
124	New Zealand	HI	Ban	B	Yes	Yes	Yes	Yes	Yes	Yes
125	Nicaragua	LMI	No-Ban	D	Yes	Yes	Yes	Yes	Yes	Yes
126	Niger	LI	No-Ban	D	Yes	Yes	No	Yes	Yes	No
127	Nigeria	LMI	No-Ban	B	Yes	Yes	No	Yes	Yes	Yes
128	North Korea	LI	No-Ban	D	Yes	No	No	Yes	Yes	Yes
129	North Macedonia	UMI	Ban	A	Yes	Yes	Yes	Yes	Yes	No
130	Norway	HI	Ban	B	Yes	Yes	Yes	Yes	Yes	Yes
131	Oman	HI	Ban	B	Yes	Yes	Yes	Yes	Yes	Yes
132	Pakistan	LMI	No-Ban	B	Yes	Yes	No	Yes	Yes	Yes
133	Palau	HI	No-Ban	B	No	Yes	No	No	No	No
134	Panama	HI	No-Ban	C	Yes	Yes	Yes	Yes	Yes	Yes
135	Papua New Guinea	LMI	No-Ban	C	No	Yes	No	Yes	Yes	Yes
136	Paraguay	UMI	No-Ban	C	Yes	Yes	Yes	Yes	Yes	No
137	Peru	UMI	No-Ban	B	Yes	Yes	Yes	Yes	Yes	Yes
138	Philippines	LMI	No-Ban	A	Yes	Yes	Yes	Yes	Yes	Yes
139	Poland	HI	Ban	B	Yes	Yes	Yes	Yes	Yes	Yes
140	Portugal	HI	Ban	B	Yes	Yes	Yes	Yes	Yes	Yes
141	Qatar	HI	Ban	B	Yes	Yes	No	Yes	Yes	Yes
142	Romania	HI	Ban	B	Yes	Yes	Yes	Yes	Yes	Yes
143	Russia	UMI	No-Ban	B	Yes	Yes	No	Yes	Yes	Yes
144	Rwanda	LI	No-Ban	B	Yes	Yes	No	Yes	Yes	Yes
145	Saint Kitts and Nevis	HI	No-Ban	C	No	Yes	No	No	No	No
146	Saint Lucia	UMI	No-Ban	C	No	Yes	Yes	Yes	Yes	No
147	Saint Vincent and Grenadines	UMI	No-Ban	D	Yes	Yes	No	Yes	No	No
148	Samoa	UMI	No-Ban	B	Yes	Yes	No	Yes	Yes	No
149	San Marino	HI	No-Ban	B	No	Yes	No	No	No	No
150	São Tomé and Príncipe	LMI	No-Ban	C	No	Yes	No	Yes	Yes	No
151	Saudi Arabia	HI	Ban	B	Yes	Yes	Yes	Yes	Yes	Yes
152	Senegal	LMI	No-Ban	C	Yes	Yes	No	Yes	Yes	Yes
153	Serbia	UMI	Ban	B	Yes	Yes	Yes	Yes	Yes	Yes
154	Seychelles	HI	Ban	C	Yes	Yes	No	Yes	Yes	No
155	Sierra Leone	LI	No-Ban	D	Yes	Yes	No	Yes	Yes	No
156	Singapore	HI	No-Ban	B	Yes	Yes	Yes	Yes	Yes	Yes
157	Slovakia	HI	Ban	B	Yes	Yes	Yes	Yes	Yes	Yes
158	Slovenia	HI	Ban	B	Yes	Yes	Yes	Yes	Yes	Yes
159	Solomon Islands	LMI	No-Ban	B	No	Yes	No	Yes	Yes	No
160	Somalia	LI	No-Ban	D	No	No	No	Yes	Yes	Yes
161	South Africa	UMI	Ban	B	Yes	Yes	Yes	Yes	Yes	Yes
162	South Korea	HI	Ban	B	Yes	Yes	Yes	Yes	Yes	Yes
163	South Sudan	LI	No-Ban	B	No	Yes	No	Yes	Yes	Yes
164	Spain	HI	Ban	B	Yes	Yes	Yes	Yes	Yes	Yes
165	Sri Lanka	LMI	No-Ban	B	Yes	Yes	Yes	Yes	Yes	Yes
166	Sudan	LI	No-Ban	C	Yes	Yes	No	Yes	Yes	Yes
167	Suriname	UMI	No-Ban	D	Yes	Yes	Yes	Yes	Yes	No
168	Sweden	HI	Ban	B	Yes	Yes	Yes	Yes	Yes	Yes
169	Switzerland	HI	Ban	B	Yes	Yes	Yes	Yes	Yes	Yes
170	Syria	LI	No-Ban	C	Yes	Yes	No	Yes	Yes	Yes
171	Taiwan ^1^	HI	Ban	B	Yes	No	No	Yes	No	No
172	Tajikistan	LI	No-Ban	B	Yes	Yes	No	Yes	Yes	No
173	Tanzania	LMI	No-Ban	C	Yes	Yes	No	Yes	Yes	No
174	Thailand	UMI	No-Ban	B	Yes	Yes	Yes	Yes	Yes	Yes
175	Timor-Leste	LMI	No-Ban	B	No	Yes	No	Yes	Yes	No
176	Togo	LI	No-Ban	C	Yes	Yes	No	Yes	Yes	Yes
177	Tonga	UMI	No-Ban	B	No	Yes	No	Yes	Yes	No
178	Trinidad and Tobago	HI	No-Ban	C	Yes	Yes	Yes	Yes	Yes	No
179	Tunisia	LMI	No-Ban	C	Yes	Yes	Yes	Yes	Yes	Yes
180	Turkey	UMI	Ban	B	Yes	Yes	Yes	Yes	Yes	Yes
181	Turkmenistan	UMI	No-Ban	D	Yes	Yes	No	Yes	Yes	Yes
182	Tuvalu	UMI	No-Ban	B	No	Yes	No	No	No	No
183	Uganda	LI	No-Ban	B	Yes	Yes	No	Yes	Yes	Yes
184	Ukraine	LMI	No-Ban	B	Yes	Yes	No	Yes	Yes	Yes
185	United Arab Emirates	HI	No-Ban	B	Yes	Yes	No	Yes	Yes	Yes
186	United Kingdom	HI	Ban	B	Yes	Yes	Yes	Yes	Yes	Yes
187	United States of America	HI	No-Ban	B	Yes	Yes	Yes	Yes	Yes	Yes
188	Uruguay	HI	Ban	C	Yes	Yes	Yes	Yes	Yes	Yes
189	Uzbekistan	LMI	No-Ban	C	Yes	Yes	Yes	Yes	Yes	Yes
190	Vanuatu	LMI	No-Ban	B	Yes	Yes	No	Yes	Yes	No
191	Venezuela	UMI	No-Ban	B	Yes	Yes	Yes	Yes	Yes	Yes
192	Vietnam	LMI	No-Ban	A	Yes	Yes	No	Yes	Yes	Yes
193	Yemen	LI	No-Ban	D	Yes	Yes	No	Yes	Yes	Yes
194	Zambia	LMI	No-Ban	C	Yes	Yes	No	Yes	Yes	Yes
195	Zimbabwe	LMI	No-Ban	B	Yes	Yes	No	Yes	Yes	Yes
	Total (Yes)				165	179	96	186	178	134

^1^ 193 UN Member States plus two regional entities (Taiwan and Hong Kong) are included in this list. Note that not all databases listed here contain data for the listed countries; ^2^ World Bank Income Groups as per Fiscal Year 2021: HI: High Income; UMI: Upper-Middle Income; LMI: Lower-Middle Income; LI: Low Income; ^3^ Asbestos bans as reported by the International Ban Asbestos Secretariat July 2019 update; ^4^ NAP country categories are: A (country that has bona fide NAP), B (country that does not have bona fide NAP but have proxy NAP), C (country that has neither bona fide or proxy NAP but has relevant published information), D (country that has no relevant information). See text for definition of document category; ^5^ USGS: United States Geological Survey; Asbestos Statistics and Information. Availability of data for 1920–2017 used; ^6^ UN Comtrade: UN International Trade Statistics Database. Tabulated for availability of data for asbestos containing materials, Comtrade code 681140 (asbestos cement; articles thereof; years available 2017–2019) or Comtrade code 6812 (fabricated asbestos fibres; mixtures with a basis of asbestos or of asbestos and magnesium carbonate; articles of such mixtures or of asbestos; years available 1996–2018) or Comtrade code 681320 (friction material and articles thereof not mounted; for brakes, clutches or the like, with a basis of asbestos; years available 2007–2018). Availability of data for timeframe 1996–2019 used; ^7^ WHO MBD: WHO Mortality Database. Availability of data for ICD-10 Code C45 Mesothelioma or ICD-10 Code J61 Pneumoconiosis due to asbestos and other mineral fibres (asbestosis). Availability of data for timeframe 1994-2017 used; ^8^ WHO GBD: WHO Global Burden of Disease studies. Availability of data for 2017 used; ^9^ WHO GHE: WHO Global Health Estimates. Availability of data for 2016 used. Countries with poor quality data were counted as countries with available data. See GHE website for description of poor-quality data; ^10^ WHO GCO: WHO Global Cancer Observatory. Availability of data for 2018 used. Note that historical data not available due to difference in estimation method.

**Table A3:** Availability of Data in International and National Sources in Relation to Each NAP Item.

Theme	Items of National Asbestos Profile	International Sources						National Sources, etc.
		*Asbestos Data*		*Disease Data*				
		Consumption of Raw Asbestos (USGS ^1^)	Asbestos-Containing Material (UN Comtrade ^2^)	Reported Mortality of Mesothelioma or Asbestosis (WHO MDB ^3^)	Estimated Incidence of Mesothelioma (GBD ^4^)	Estimated Mortality of Mesothelioma (WHO GHE ^5^)	Reported or estimated Mortality of Mesothelioma (WHO GCO ^6^)	
Legislation	I-1. *Current regulations on the different forms of asbestos* I-15. *National enforceable occupational exposure limits for chrysotile asbestos* I-16. *The system for inspection and enforcement of the exposure limits*							I-1, I-15, I-16: Existing government laws and regulations
Asbestos	I-2. *Import and consumption of asbestos per year (total and per major uses and forms)* I-3. *Import of asbestos-containing materials* I-4. *Domestic production of asbestos (if applicable)* I-5. *Domestic production of asbestos-containing materials*	I-2, I-4	I-3, I-5					I-2, I-3: National trade statistics; I-4: National mining statistics; I-5: National manufacturing statistics
Diseases	I-9. *Estimate of the burden of diseases related to asbestos: disability adjusted life years and deaths attributable to asbestos exposure* I-10. *Prevalence of asbestosis – national data, a breakdown by industries if available* I-11. *Incidence of lung cancer among workers exposed to asbestos* I-12. *Incidence of mesothelioma* I-17. *Estimated economic losses due to asbestos-related diseases* I-18. *Major studies on epidemiology of asbestos-related diseases in the country*			I-9 (reported deaths)	I-9, I-10, I-11, I-12 deaths, DALY)	I-9, I-12 (estimated	I-9, I-12 (reported a/o estimated deaths)	I-10: National compensation statistics; I-12: National/Regional Cancer Registry data; I-18: PUBMED
Risk Assessment	I-6. *Estimated total number of workers exposed to asbestos in the country* I-7. *Full list of industries where exposure to asbestos is present in the country and list of industries with the largest numbers of workers potentially exposed to asbestos* I-8. *Industries with high risk of exposure (where overexposure is documented as exceeding occupational exposure limits) and estimated total number of workers at high risk* I-13. *Estimates on the percentage of house stock and vehicle fleet containing asbestos* I-14. *Total number of workers eligible for compensation for asbestos-related diseases, such as asbestosis, lung cancer and mesothelioma (per year) and the numbers of individuals compensated yearly*							I-6, I-8: Industrial hygiene or occupational health data; I-7, I-13: Specific industry inventory; I-14: Occupational disease compensation data.

^1^ United States Geological Survey, Asbestos Statistics and Information. Availability of data for 1920–2017 used; ^2^ UN International Trade Statistics Database. Tabulated for availability of data for asbestos containing materials, Comtrade code 681140 (asbestos cement; articles thereof; years available 2017–2019) or Comtrade code 6812 (fabricated asbestos fibres; mixtures with a basis of asbestos or of asbestos and magnesium carbonate; articles of such mixtures or of asbestos; years available 1996–2018) or Comtrade code 681320 (friction material and articles thereof not mounted; for brakes, clutches or the like, with a basis of asbestos; years available 2007–2018). Availability of data for timeframe 1996–2019 used; ^3^ WHO Mortality Database. Availability of data for ICD-10 Code C45 Mesothelioma or ICD-10 Code J61 Pneumoconiosis due to asbestos and other mineral fibres (asbestosis). Availability of data for timeframe 1994–2017 used; ^4^ Global Burden of Disease studies. Availability of data for 2017 used; ^5^ WHO Global Health Estimates. Availability of data for 2016 used. Countries with poor quality data were counted as countries with available data. See GHE website for description of poor-quality data; ^6^ WHO Global Cancer Observatory. Availability of data for 2018 used. Note that historical data not available due to difference in estimation method.
